# Abrasive Enamel and Dentin Wear Resulting from Brushing with Toothpastes with Highly Discrepant Relative Enamel Abrasivity (REA) and Relative Dentin Abrasivity (RDA) Values

**DOI:** 10.3290/j.ohpd.b3858625

**Published:** 2023-02-02

**Authors:** Liana Dobler, Blend Hamza, Thomas Attin, Florian J. Wegehaupt

**Affiliations:** a Postgraduate Student, Clinic of Conservative and Preventive Dentistry, Center for Dental Medicine, University of Zürich, Zürich, Switzerland. Performed the experiments as part of a doctoral thesis, wrote the manuscript.; b Resident, Clinic of Orthodontics and Pediatric Dentistry, Center for Dental Medicine, University of Zürich, Zürich, Switzerland. Study idea, supervision, proofread the manuscript.; c Professor and Clinic Director, Clinic of Conservative and Preventive Dentistry, Center for Dental Medicine, University of Zürich, Zürich, Switzerland. Supervision, critical evaluation of the manuscript.; d Head Division of Preventive Dentistry and Oral Epidemiology, Clinic of Conservative and Preventive Dentistry, Center for Dental Medicine, University of Zürich, Zürich, Switzerland. Supervision, conceived and designed the experiment, proofread the manuscript.

**Keywords:** abrasive dentin wear, abrasive enamel wear, RDA, REA, toothpaste abrasivity

## Abstract

**Purpose:**

To investigate the absolute wear caused by toothpastes with highly discrepant REA (Relative Enamel Abrasivity) and RDA (Relative Dentin Abrasivity) values on both enamel and dentin: Candida Peppermint (CP; REA: 1; RDA: 42), Colgate Total Original (CTO; REA: 4; RDA: 100), Signal White System (SWS; REA: 8; RDA: 143), and Candida White Diamond (CWD; REA 244; RDA: 12).

**Materials and Methods:**

Eighty (80) bovine enamel samples and 80 dentin samples were divided into four groups each (n = 20) and investigated after a 6-h brushing procedure (21,600 cycles, 60 cycles/min, load of 2.5 N) with the four toothpastes. The abrasive enamel and dentin wear were registered using a contact profilometer. The median and interquartile range (IQR) of the abrasive enamel and dentin wear were calculated for each group. Pairwise comparisons were conducted using the Wilcoxon signed-rank exact test, and the p-value was adjusted according to Holm (statistical significance set at 0.05).

**Results:**

CWD led to the highest abrasive enamel wear (9.86 µm [5.77]). CTO caused the highest abrasive dentin wear (166.70 µm [69.90]), being statistically significantly higher than the wear for CP (54.20 µm [24.00]) and CWD (17.00 µm [7.80]) (p = 0.00001). The abrasive dentin wear for CWD was statistically significantly lower in comparison to all other groups (p = 0.00001).

**Conclusion:**

Toothpastes with highly discrepant REA and RDA values presented statistically significantly different absolute wear on enamel and dentin. REA and RDA values should both be declared for every toothpaste.

Tooth wear occurs physiologically due to the exposure of dental tissue to the environment, and it increases with age.^31^ The global prevalence of tooth wear is 46.7% and higher,^30^ and is therefore of considerable importance. Excessive toothbrushing with abrasive toothpastes and additional improper brushing techniques can lead to further dental abrasion.

A toothpaste’s abrasivity is essentially driven by the abrasive particles it contains, that is, their size, shape and concentration.^21^ Mostly, traditional toothpastes contain silica- or calcium-based abrasives and different chemical syntheses allow the adjustment of their abrasion and cleaning performance.^7^ Additionally, novel toothpastes containing diamond particles as abrasives have entered the market.

As human dentin is softer than enamel and is therefore assumed to abrade more quickly, toothpastes are tested for abrasive dentin wear by default.^10^ Abrasives in the emerging diamond toothpastes, however, show greater hardness than enamel and therefore provide the potential to abrade it.^11,33^ Nonetheless, diamond toothpastes are commonly tested for their abrasive wear on dentin too, but their abrasive wear on enamel is disregarded. Studies have stated that toothpastes containing diamond abrasive particles are more abrasive on sound enamel than are traditional toothpastes.^12,33^

In order to evaluate the REA and RDA values, it should be mentioned that, according to the International Standard Organization (ISO 11609:2017), the abrasivity of toothpastes on enamel should not be more than four times that of the primary reference material. As the primary reference material’s REA is set as 10, this results in a maximum REA of 40. For the interpretation of RDA values, Imfeld et al^19^ provided a 5-group classification: very low abrasivity (RDA 0–20), low abrasivity (RDA 20–40), medium abrasivity (RDA 40–60), high abrasivity (RDA 60–80), and very high abrasivity (RDA > 80).^19^ A modification/simplification of this classification was suggested by Hamza et al:^12^ low abrasion (RDA < 40, REA < 4), moderate abrasion (RDA 40–80, REA 4–8), and high abrasion (RDA > 80, REA > 8).

As the REA and RDA represent relative values (abrasivity relative to that caused by a standard abrasive), concrete visualisation of wear is rather difficult. Therefore, the present study aimed to investigate the absolute effect (wear) of toothpastes with highly different REA and RDA values on both enamel and dentin. The first null hypothesis was that there is no difference in the wear of enamel when brushed with toothpastes with highly different REA values. The second null hypothesis was that there is no difference in the wear of dentin when brushed with toothpastes with highly different RDA values. The third null hypothesis was that toothpastes with low RDA values will also cause the lowest enamel wear.

## Materials and Methods

### Sample Preparation and Allocation

For this study, 80 enamel and dentin samples each (total of 160 samples) were prepared from 20 extracted permanent bovine mandibular incisors (4 enamel and 4 dentin samples per tooth). Using a trephine drill with an inner diameter of 3 mm, four samples were milled out of the crown (enamel samples: A–D) and the root (dentin samples: E–H) of each tooth. Since these drilled-out samples were too delicate for the experimental equipment, they had to be embedded in acrylic resin (Paladur, Haraeus Kulzer; Hanau, Germany). For this purpose, the enamel and dentin samples were placed in a prefabricated silicone mold with the surface to be examined facing downward. By filling the silicone mold with acrylic resin, it was possible to produce approximately 5.7-mm-high samples with a diameter of 6 mm. To let the acrylic resin polymerize entirely, the filled silicone mold was placed inside a laboratory incubator (Palamat elite, Heraeus Kulzer) at 45°C and 2 bar for 10 min. The cured samples were milled from the bottom to a common approximate height using a cross-cut milling machine. This was followed by grinding the enamel or dentin surfaces flat using a grinding and polishing machine (Tegramin-30, Struers; Ballerup, Denmark). Enamel surfaces were ground under constant water cooling using 1200-, 2000-, and 4000-grit silicon-carbide papers (SiC paper, Struers), for 10, 20, and 30 s, respectively, at a turntable speed of 150 rpm in synchronization and a load of 5 N. Dentin surfaces were ground under the same conditions using only 2000- and 4000-grit silicon-carbide papers for 15 and 30 s, respectively. Subsequently, the samples were again machined from the bottom with the cross-cut mill to the required height of 3 mm. Finally, two parallel lines serving as reference notches for the profilometric analysis were scratched into each sample’s surface using a sharp metal pen held in a custom-made device. These lines were located in the acrylic resin part of the sample surface as close to the tooth substance as possible.

**Fig 1:**
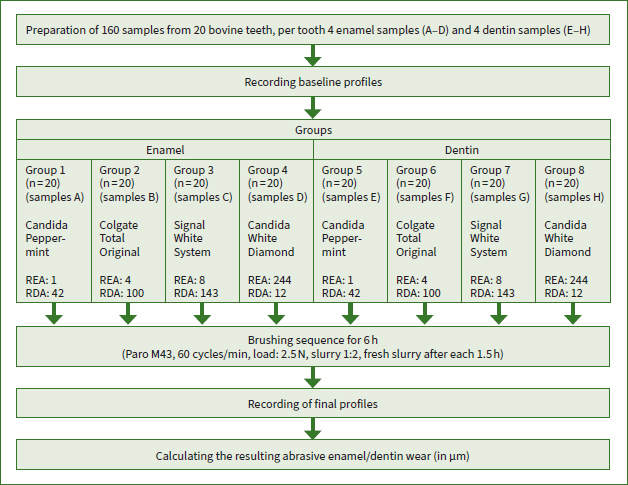
Study design.

The enamel and dentin samples were then allocated into the eight groups (1–4 for enamel and 5–8 for dentin, n = 20). All “A” samples were allocated to group 1, all “B” samples to group 2, etc. Therefore, each group contained one sample from the same incisor crown or root. Until further use, the samples were constantly stored in tap water. Figure 1 shows a summary of the study design.

### Brushing Procedure

**Fig 2:**
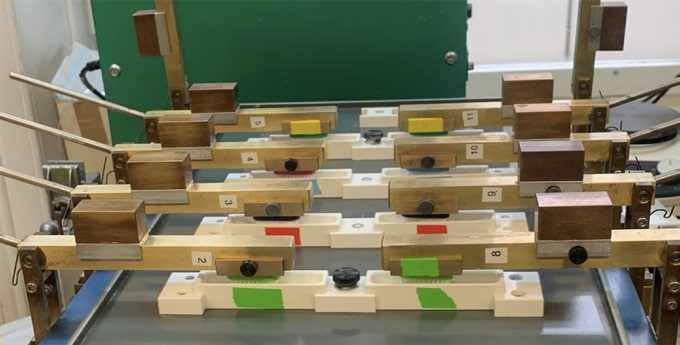
Experimental set-up in the brushing machine.

The brushing of the samples was performed in an automatic brushing machine for a period of six hours. In each brushing container of the brushing machine, two samples from the same group were placed. Parts of the sample surfaces were covered with adhesive tape for fixation and protection against abrasion during the later brushing. The areas thus covered served as reference areas and, together with the reference notches, ensured subsequent superimposition of the baseline and post-brushing profiles. Toothpaste slurries for use in the automatic brushing machine were prepared at a ratio of one part toothpaste by weight to two parts artificial saliva by weight. The artificial saliva was prepared according to Klimek et al.^20^ Four toothpastes with highly different REA and RDA values were used. Brushing in groups 1 and 5 was performed using a slurry of Candida Peppermint (REA: 1; RDA: 42; Mibelle; Buchs, Switzerland), groups 2 and 6 using a slurry of Colgate Total Original (REA: 4; RDA: 100; Colgate-Palmolive; Swidnica, Poland), groups 3 and 7 using a slurry of Signal White System (REA: 8; RDA: 143; Unilever Switzerland; Thayngen, Switzerland), and groups 3 and 8 using a slurry of Candida White Diamond (REA: 244; RDA 12, Mibelle). Table 1 shows the composition of all toothpastes used (manufacturers’ information) and their identified REA and RDA according to Hamza et al.^12^ The brushing containers were filled with 3 ml of the respective slurry. A medium-bristled standard toothbrush (Paro M43, Esro; Kilchberg, Switzerland) was used at 2.5 N brushing force and 60 cycles/min brushing speed. Every 1.5 h (i.e. 5400 brushing cycles), the slurries were replaced. Figure 2 shows the experimental set-up in the brushing machine.

**Table 1:** Composition of the tested toothpastes according to the manufacturer

Tested toothpastes (manufacturer)	REA/RDA	Composition
Candida Peppermint (Mibelle; Buchs, Switzerland)	REA: 1 RDA: 42	Dicalcium phosphate dihydrate, glycerin, aqua, hydrated silica, sodium lauryl sulfate, sodium monofluorophosphate (1490 ppm), paraffinum liquidum, aroma, cellulose gum, panthenol, sodium saccharin, bisabolol, limonene, linalool
Colgate Total Original (Colgate-Palmolive; Swidnica, Poland)	REA: 4 RDA: 100	Glycerin, aqua, hydrated silica, sodium lauryl sulfate, arginine, aroma, cellulose gum, zinc oxide, benzyl alcohol, poloxamer 407, zinc citrate, tetrasodium pyrophosphate, xanthan gum, cocamidopropyl betaine, sodium fluoride (1450 ppm), sodium saccharin, phosphoric acid, sucralose, Cl 77891
Signal White System (Unilever Switzerland; Thayngen, Switzerland)	REA: 8 RDA: 143	Calcium carbonate, aqua, hydrogenated starch hydrolysate, hydrated silica, sodium lauryl sulfate, sodium monofluorophosphate (1450 ppm), aroma, cellulose gum, benzyl alcohol, trisodium phosphate, sodium saccharin, glycerin, sodium laureth sulfate, CI 74160
Candida White Diamond (Mibelle)	REA: 244 RDA: 12	Aqua, hydrogenated starch hydrosylate, potassium citrate, hydrated silica, sodium lauryl sulfate, xanthan gum, aroma, sodium acrylates/C10-30 alkyl acrylate crosspolymer, sodium fluoride (1450 ppm), sodium saccharin, zinc chloride, diamond powder, methlyparaben, allantoin, limonene, linalool, benzyl alcohol, CI 77891

REA/RDA data according to Hamza et al.^12^

### Profilometric Measurement

Before starting the brushing procedure, baseline profiles of the sample surfaces were recorded using a contact profilometer (Perthometer S2, Mahr; Göttingen, Germany).^3^ To provide correct positioning and enable repositioning of each sample in the profilometer, a prefabricated jig was used. Per sample, five profiles with a lateral distance of 250 µm were recorded perpendicular to the brushing direction. Recording of profiles started on the first reference notch, passing the brushed test area, and ended on the second reference notch, ensuring two reference areas on both sides of the brushed test area for later superimposition of baseline and post-brushing profiles. The samples were kept wet during the measurement process to prevent deformation of the sample surfaces due to desiccation. Having undergone the entire 6-h brushing program, the samples were rinsed with tap water, the adhesive tape was removed and final profiles were registered. In the next step, the baseline profiles and the respective final profiles were superimposed with custom-designed software, and the difference between the baseline profile and final profile was considered as the resulting abrasive wear of enamel or dentin. As determined in a previous study, the profilometer used had a minimum measurement limit of 0.105 µm.^3^

### Statistical Analysis

Median and interquartile range (IQR) of the abrasive enamel and dentin wear were calculated for each group. Pairwise comparisons between the groups (enamel groups 1–4 and dentin groups 5–8) were conducted using the Wilcoxon signed-rank exact test, and the p-value was adjusted according to Holm. The significance level was set at 0.05. All statistical analyses were computed with the statistical software R25, including the package “tidyverse”.^35^

## Results

### Enamel

Table 2 depicts the resulting abrasive enamel wear for each group. Candida White Diamond led to the highest abrasive enamel wear (median/IQR; 9.86/5.77 µm), being statistically significantly higher than the wear in all other groups (p = 0.00001). Colgate Total Original caused the second highest abrasive enamel wear (0.19/0.13 µm), showing a statistically significant difference vs Signal White System (p = 0.04) and Candida Peppermint (p = 0.0005). Candida Peppermint (0.08/0.04 µm) and Signal White System (0.08/0.11 µm) presented almost equally low abrasive enamel wear, showing no statistically significant difference (p = 0.39).

**Table 2:** Median, IQR, minimum and maximum of the abrasive enamel wear (in µm) in the tested groups plus REA values (Hamza et al^12^)

Abrasive enamel wear [µm]					
Group	REA	Median	IQR	Minimum	Maximum
Candida Peppermint	REA: 1	0.08 (A)	0.04	-0.05	0.20
Colgate Total Original	REA: 4	0.19 (B)	0.13	0.00	0.42
Signal White System	REA: 8	0.08 (A)	0.11	0.00	0.29
Candida White Diamond	REA: 244	9.86 (C)	5.77	5.67	23.03

Same letters after the median value indicate no statistical significance among the groups.

### Dentin

The abrasive dentin wear of all groups is presented in Table 3. Colgate Total Original caused the highest abrasive dentin wear (median/IQR; 166.70/69.90 µm), being statistically significantly higher than the wear for Candida Peppermint (54.20/24.00 µm) and Candida White Diamond (17.00/7.80 µm) (p = 0.00001). Signal White System showed the second highest abrasive dentin wear (151.50/37.80 µm) and was not statistically significantly different from Colgate Total Original (p = 0.5). The abrasive wear for Candida White Diamond was statistically significantly lower in comparison to all other groups (p = 0.00001).

**Table 3:** Median, IQR, minimum and maximum of the abrasive dentin wear (in µm) in the tested groups plus RDA values (from publication by Hamza et al^12^)

Abrasive dentin wear [µm]					
Group	RDA	Median	IQR	Minimum	Maximum
Candida Peppermint	RDA: 42	54.20 (A)	24.00	28.40	100.50
Colgate Total Original	RDA: 100	166.70 (B)	69.90	93.40	220.80
Signal White System	RDA: 143	151.50 (B)	37.80	97.30	216.60
Candida White Diamond	RDA: 12	17.00 (C)	7.80	10.30	32.20

Same letters after the median value indicate no statistical significance among the groups.

## Discussion

The aim of this in-vitro study was to investigate the abrasive wear of four toothpastes with highly discrepant REA and RDA values on dental enamel and dentin. In several cases, RDA values are indicated by the manufacturer for their toothpastes and are intended to serve as a guide for the consumer. Especially novel toothpastes with diamond abrasives advertise low RDA values, without information about their impact on the enamel. Due to the fact that dentin is softer than enamel and is therefore more susceptible to substance loss during brushing, the abrasion of dentin might have been viewed more critically than enamel. Abrasives used in earlier toothpastes showed lower hardness than enamel, which consequently led to the assumption that they are unable to abrade it.^16^ This might be another reason for the neglect of REA values. Nonetheless, diamond powder contained in novel toothpastes as abrasives shows much higher hardness than enamel and is therefore able to abrade it.^33^ Therefore, low RDA values of diamond toothpastes could lead to the assumption that they are extremely gentle on teeth. However, manufacturers’ studies must have only been conducted on dentin to provide RDA results, without taking enamel into consideration. This study and others^12,33^ showed that the use of diamond toothpastes (i.e. Candida White Diamond) leads to high abrasive wear on sound enamel in comparison to traditional toothpastes. Therefore, not only for novel toothpastes would it be helpful to know their impact on the chiefly brushed enamel and the exposed dentin, if any, but also for every other conventional toothpaste. This means that REA and RDA values should be provided by manufacturers. Regarding this aspect, in 2019, a petition was submitted to the European Parliament demanding the obligatory information on the abrasiveness of toothpastes.^8^

### Experimental setup

To evaluate abrasive dentin and enamel wear in this study, bovine teeth were used. Bovine dental hard tissues have been proven to be suitable alternatives to human teeth in abrasion studies^4,5,34^ due to similar physical and chemical properties.^1^ Bovine teeth are more easily available in large numbers, with similar (surface-) texture and in better condition than human teeth.^37^ As such, bovine teeth provide a large surface area not only on the crown as but also on the root, and several samples of enamel (from the crown) and dentin (from the root) can be gained from the same tooth.^32^ This allows a certain homogeneity and high comparability of results.^36^ Attin et al^5^ proved that no difference exists between human and bovine enamel regarding abrasive wear only. Moreover, for studies on dentin, it was also found that mandibular bovine incisors are suitable for abrasion studies in general,^32^ and RDA studies in particular.^34^

Profilometric analysis was performed using a contact profilometer. In numerous earlier studies, this method was used to register loss of dental hard tissue after brushing procedures.^2,9,18,22^ Providing a minimum measurement limit of 0.105 µm and an approximate reproducibility of 40 nm, measurements obtained with this method are considered quite accurate.^3^ Furthermore, the profilometric measurement used here contributes to the comparison of the direct abrasive wear vs the indirect radiotracer method used for relative enamel or dentin abrasivity.^10^ However, it must be borne in mind that the stylus tip can cause soft materials (e.g. dentin) to deform or scratch/indent the surface. A study by Paepegaey et al^23^ investigated exactly those contact-profilometer-induced scratches and found that scratch depth was below 1 µm, which makes an effect on study conclusions unlikely. Furthermore, even if the stylus tip caused a deformation of the dentin, which might result in overestimation of the wear, this would be uniform in all dentin groups. Additionally, it can be assumed that due to the measuring set-up with baseline and final profiles, such a deformation of the dentin surface is negligible, as the deformation would occur during both recordings and therefore be eliminated during the later superimposition of the profiles. Nevertheless, alternative measuring techniques such as non-contact laser profilometry or confocal laser scanning microscopy were found to be equally suitable in erosion studies and could be considered for further abrasion studies.^23,27^

For the determination of REA and RDA values, the radiotracer method is still considered the “gold standard”.^10,16^ This method, however, provides relative values with variance up to 20%.^10^ It was shown that similar results for abrasivity measurements can be provided by both the traditional radiotracer method and the profilometric method.^26,28^ Additionally, RDA measurements using profilometry showed reproducibility and differentiation at least as good as the radiotracer method, and that human and bovine teeth could be used interchangeably.^29^ However, discrepancies between the two methods can be found in the literature^10,24^ and should be considered especially in the higher abrasivity range.^24^

As acid immersion or EDTA would have been needed to remove the smear layer resulting from preparatory grinding, this was not performed. The use of acids or EDTA would have affected both enamel and dentin surfaces, which would have resulted in increased susceptibility of the substrates to abrasion. Furthermore, as all samples were prepared equally , any protective properies possibly provided by the smear layer would apply equally to both enamel and dentin samples.

In this study, samples were brushed with a load of 2.5 N, as in the study by Wiegand and Attin,^36^ who applied a 2 to 3 N brushing load for abrasion studies. Brushing was performed for 6 h, i.e. 21,600 cycles (43,200 brushing strokes). Following the recommendations to brush teeth two to three times a day, this represents an actual brushing time of approximately four years according to the assumption of Wiegand and Attin^36^ of 10 brushing strokes per tooth per brushing session.

During the brushing procedure, the containers held 3 ml of the respective slurry, which was replaced every 1.5 h. It might be argued that the abrasion intensity could vary from the start to the end of the 1.5 h due to exsiccation of the slurry. The replacement of the slurry every 1.5 h – and not longer – should prevent the samples from being brushed with slurries low in lubricant.

As a limitation, it must again be mentioned that this in-vitro study is purely laboratory-based. Factors that contribute to the natural protection of dental tissue, such as protective properties of the saliva (e.g. pellicle formation, hard tissue remineralisation)^6^ or other influences, such as demineralisation due to matured biofilm or intake of erosive foodstuffs and beverages, were not investigated. As the values obtained here were only compared within the present study itself, and not transferred to the clinical situation, a possible over- or underestimation of the wear values due to not simulating all possible clinical factors that could influence wear, seems to be acceptable.

### Abrasive Enamel and Dentin Wear

Our first and second null hypotheses must be rejected, as brushing with the toothpastes of highly diverse REA and RDA values resulted in significantly different enamel and dentin wear. Also, the third null hypothesis that toothpastes with low RDA values, therefore causing the lowest dentin wear, would also cause the lowest enamel wear must be rejected, as Candida White Diamond – with the lowest dentin wear – caused the highest enamel wear.

Candida White Diamond showed the statistically significantly highest abrasive enamel wear in comparison to all other toothpastes used in this study. It was the only toothpaste containing diamond powder as the abrasive. This finding can probably be attributed to the fact that diamond particles exhibit much higher hardness than dental enamel,^12^ which agrees with the high REA reported for this toothpaste by Hamza et al.^12^ By way of illustration, imagine rubbing diamond particles on rubber; the rubber will give way, just as dentin would: hard diamond particles sink through or deform the soft dentin rather than cutting it. In contrast, rubbing diamond particles on glass will scratch and abrade the unyielding glass (enamel).^12^

Taking into consideration that in a healthy dentition enamel is the dental tissue chiefly exposed to the toothpaste, so that REA values should be investigated and acknowledged. After all, from a preventive perspective, the enamel should be preserved and protected as much as possible, instead of being unintentionally brushed away or abraded through the use of supposedly harmless high-RDA diamond toothpastes. Based on the results of this study, at worst, the use of Candida White Diamond would lead to a yearly enamel loss of about 5.76 µm (maximum enamel wear 23.03 µm/4 years). Consequently, provoking an enamel loss of 1 mm would take around 170 years. This remarkable loss could be considered harmless in terms of preserving a certain enamel thickness over a lifetime, never abrading down to the dentin. Nonetheless, other parameters associated with clinical toothbrushing that affect dental abrasivity, such as the brushing force,^15^ toothbrush type (traditional or sonic)^15^ and toothbrush characteristics (stiffness,^15^ bristle arrangement^13^ etc) should be considered. Hamza et al^15^ stated that use of a sonic toothbrush in combination with toothpastes with high abrasivity could lead to higher abrasive dentin wear. Moreover, the mineral quality of the enamel should be examined. A study by Wegehaupt et al^33^ found that a diamond toothpaste – namely Candida White Diamond – provoked higher abrasive wear on previously eroded enamel than on sound enamel.^33^ Taking into consideration that these factors might increase the abrasive wear of enamel unintentionally and uncontrollably, an increased wear of enamel due to the use of high REA toothpastes should be avoided.

Although Colgate Total Original was recently reported to have the second highest RDA (100) in this set-up,^12^ it led to the highest abrasive dentin wear in the present study, followed closely by Signal White System (RDA: 143). Candida White Diamond with its reported RDA of 12 resulted in the lowest abrasive dentin wear, and therefore fulfilled expectations in this study. The diamond-loaded toothpaste produced hardly any damage on the dentin.

As long as no dentin is exposed, the main substrate being brushed is enamel, which should be preserved with low REA toothpastes. Pragmatically, RDA values (high or low) are less relevant in this case, as dentin is not abraded. Nevertheless, toothpastes with low RDA values should be preferred. Concretely, using a toothpaste with a high REA and low RDA under healthy conditions does not prove advantageous, since dentin is not exposed and the low RDA is thus irrelevant. However, the enamel is affected by the high REA. If a patient desires to use a toothpaste with a high REA or if a dentist wants to recommend it, it should be taken into consideration that diamond toothpastes are gentle on dentin. However, that decision should be made in consultation with a dentist, who should point out that the influence on enamel is important. Additionally, it should be mentioned that not only diamond toothpastes can provide low RDA values, as other conventional toothpastes with rather low RDA values exist.^12^

Conversely, assuming exposed dentin surfaces, the recommendation to use a toothpaste with low RDA for dentin protection seems obvious. In combination with low REA values, ideal protection for enamel could also be provided. These deliberations again point to the need for mandatory declaration of both REA and RDA values.

The cleaning efficacy of the toothpastes used here, with their highly discrepant REA and RDA values, was not investigated in this study. However, a study about dentin abrasivity and cleaning efficacy found that toothpastes which cause higher abrasive wear do not necessarily provide greater cleaning efficacy.^14^

## Conclusions

Within the limitations of this in-vitro study, it is concluded that REA and RDA values should both be considered for every toothpaste. Especially REA should be declared, as under healthy conditions the enamel is primarily exposed to the toothpaste and the first substrate to be abraded. Although RDA values are often provided to inform the consumer, REA values should be declared equally. Investigating toothpastes with highly discrepant REA and RDA values, this study demonstrated differences in the abrasivity of enamel and dentin. Obviously, dentin can be protected with the use of low-RDA toothpastes; enamel should be equally protected.
